# The haustorial transcriptome of the cucurbit pathogen *Podosphaera xanthii* reveals new insights into the biotrophy and pathogenesis of powdery mildew fungi

**DOI:** 10.1186/s12864-019-5938-0

**Published:** 2019-07-04

**Authors:** Álvaro Polonio, Pedro Seoane, M. Gonzalo Claros, Alejandro Pérez-García

**Affiliations:** 1 Departamento de Microbiología, Facultad de Ciencias, Universidad de Málaga, Bulevar Louis Pasteur 31, 29071 Málaga, Spain; 2 Instituto de Hortofruticultura Subtropical y Mediterránea “La Mayora”, Universidad de Málaga, Consejo Superior de Investigaciones Científicas (IHSM−UMA−CSIC), Bulevar Louis Pasteur 31, 29071 Málaga, Spain; 3 Departamento de Biología Molecular y Bioquímica, Facultad de Ciencias, Universidad de Málaga, Bulevar Louis Pasteur 31, 29071 Málaga, Spain

**Keywords:** Powdery mildew fungi, *Podosphaera xanthii*, Haustorium, Massive-scale RNA sequencing, Secretome, Protein structure modeling

## Abstract

**Background:**

*Podosphaera xanthii* is the main causal agent of powdery mildew disease in cucurbits and is responsible for important yield losses in these crops worldwide. Powdery mildew fungi are obligate biotrophs. In these parasites, biotrophy is determined by the presence of haustoria, which are specialized structures of parasitism developed by these fungi for the acquisition of nutrients and the delivery of effectors. Detailed molecular studies of powdery mildew haustoria are scarce due mainly to difficulties in their isolation. Therefore, their analysis is considered an important challenge for powdery mildew research. The aim of this work was to gain insights into powdery mildew biology by analysing the haustorial transcriptome of *P. xanthii*.

**Results:**

Prior to RNA isolation and massive-scale mRNA sequencing, a flow cytometric approach was developed to isolate *P. xanthii* haustoria free of visible contaminants. Next, several commercial kits were used to isolate total RNA and to construct the cDNA and Illumina libraries that were finally sequenced by the Illumina NextSeq system. Using this approach, the maximum amount of information from low-quality RNA that could be obtained was used to accomplish the de novo assembly of the *P. xanthii* haustorial transcriptome. The subsequent analysis of this transcriptome and comparison with the epiphytic transcriptome allowed us to identify the importance of several biological processes for haustorial cells such as protection against reactive oxygen species, the acquisition of different nutrients and genetic regulation mediated by non-coding RNAs. In addition, we could also identify several secreted proteins expressed exclusively in haustoria such as cell adhesion proteins that have not been related to powdery mildew biology to date.

**Conclusions:**

This work provides a novel approach to study the molecular aspects of powdery mildew haustoria. In addition, the results of this study have also allowed us to identify certain previously unknown processes and proteins involved in the biology of powdery mildews that could be essential for their biotrophy and pathogenesis.

**Electronic supplementary material:**

The online version of this article (10.1186/s12864-019-5938-0) contains supplementary material, which is available to authorized users.

## Background

*Podosphaera xanthii* is a plant-pathogenic ascomycete fungus of the *Erysiphales* order that causes powdery mildew disease in cucurbits and significantly reduces the yields of cucurbit crops [1–4]. As with other powdery mildew fungi, *P. xanthii* is an obligate biotrophic parasite that depends on living host cells for growth and reproduction. In these fungi, biotrophy is determined by the development of specialized structures of parasitism termed haustoria [5], whose main putative functions are the uptake of nutrients from the plant and the release of effectors into the host cells [6], that thus play a pivotal role in both biotrophy and pathogenesis [7]. Since this fungal structure is the battlefront between the pathogen and the plant, the identification of physiological processes carried out by proteins expressed in the haustorium should provide key information to understand the singularities of powdery mildew fungi as well as to offer opportunities for the development of novel management tools.

Detailed molecular investigations of biotrophic fungal pathogens such as powdery mildews are not very abundant, mainly due to limitations in their growth (exclusively in host tissues) and difficulties with their genetic manipulation [8]. Therefore, the study of powdery mildews is considered a challenge, and many aspects of their biotrophic lifestyle and pathogenesis are not yet resolved. However, next-generation sequencing technologies combined with different computational approaches have allowed us to obtain several powdery mildew genomes corresponding to different isolates of *Blumeria graminis* f. sp. *hordei*, *B. graminis* f. sp. *tritici*, *Erysiphe necator, Golovinomyces orontii* and *Oidium neolycopersici*, which are the causal agents of barley powdery mildew, wheat powdery mildew, grape powdery mildew, *Arabidopsis* powdery mildew and tomato powdery mildew, respectively [9–17]. The availability of several powdery mildew genomes has allowed us to obtain new insights into the pathogenesis, such as genome size expansion with the proliferation of transposable elements and effector candidate genes [9, 10], and the biotrophic lifestyle, such as the identification of missing genes and metabolic pathways, that could explain the impossibility of cultivating these fungi in artificial culture media [9, 12, 13, 18]. The different genome assemblies have also allowed us to predict the number of effector candidate genes in the genome of powdery mildew fungi [9, 15, 19]. Nevertheless, the prediction of effector gene catalogues from a fungal genome is error-prone and unreliable [20], making the use of different complementary strategies necessary.

Transcriptomic analyses provide important information about those genes that are expressed during powdery mildew infection and fungal development. Moreover, these analyses allow for more accurate prediction of effector candidate genes, since short genes, often with no homology, are hard to find in genomes. Despite the fact that transcriptomes from several powdery mildew species are available [11, 21–25], specific studies on haustorial transcriptomes, which are necessary to decipher the molecular bases of biotrophy and pathogenesis in powdery mildew species, are very limited probably due to difficulties in obtaining high-quality isolations of haustoria free of contaminants. To date, only two powdery mildew haustorial transcriptomic studies have been performed, the first from haustorial enriched barley epidermal strips [24] and the second from haustorial fractions obtained from isopycnic Percoll centrifugation [23].

In the particular case of the cucurbit powdery mildew pathogen *P. xanthii,* the analysis of the epiphytic transcriptome [25] and the development of tools for functional gene analysis [6, 26], have allowed us to identify novel functions of several effector candidates and validate their role in pathogenesis [6]. Despite such advances, however, our knowledge about the biotrophic lifestyle and pathogenesis of *P. xanthii* remains incomplete because specific information about gene expression in haustoria is virtually unknown. In this study, we developed a method to isolate *P. xanthii* haustoria virtually free of debris and performed the de novo assembly of the *P. xanthii* haustorial transcriptome and a comparative analysis of haustorial and epiphytic transcriptomes. These analyses have allowed us to detect specific and important haustorial functions and to identify a new set of effector candidate genes specifically expressed in haustoria. Our findings provide novel information about the biotrophic lifestyle and pathogenesis of *P. xanthii* and identify previously unknown aspects of powdery mildew biology.

## Results

### Flow cytometry allows the isolation of haustoria free of contaminants

**Fig. 1:**
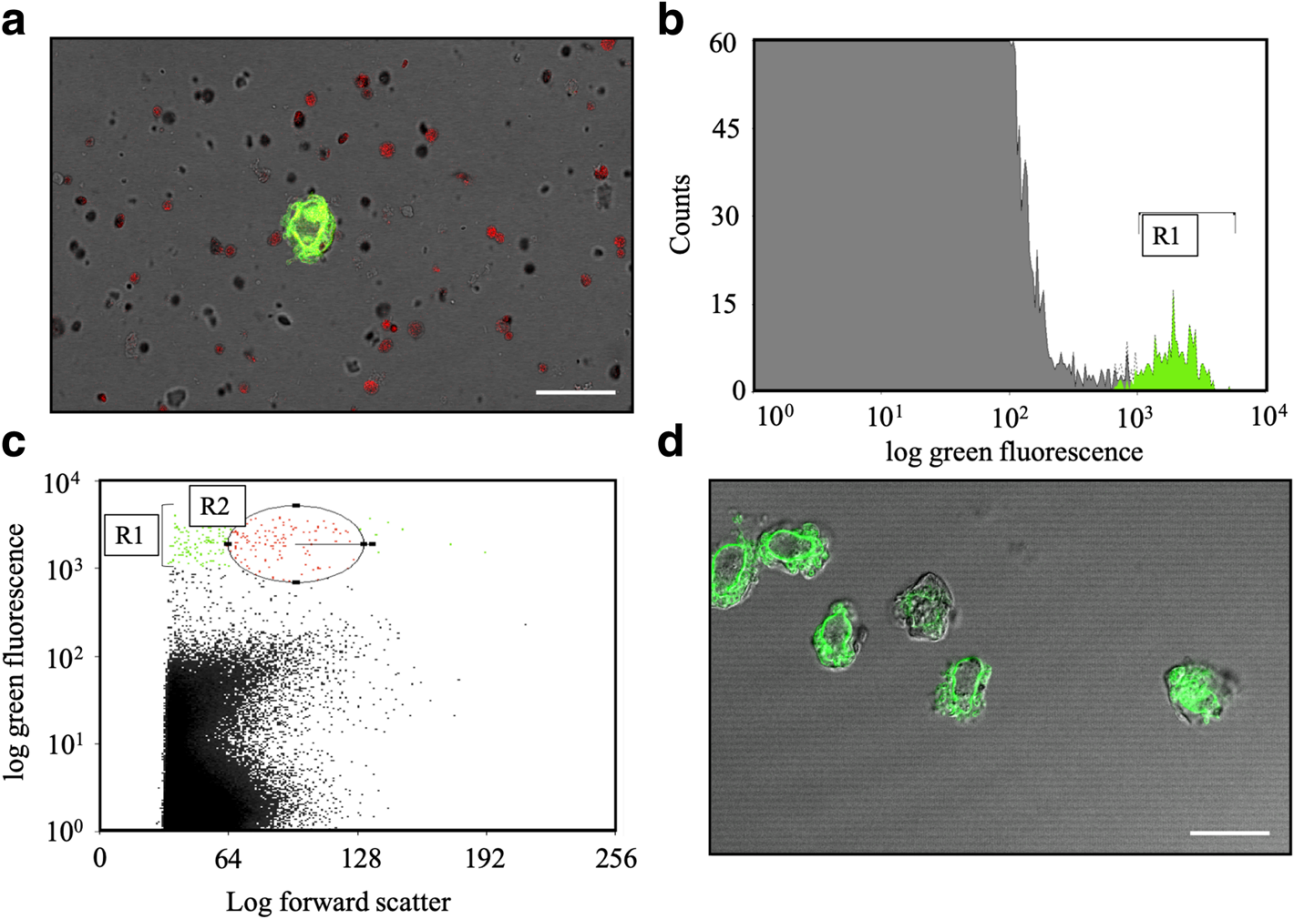
Isolation of *Podosphaera xanthii* haustorial cells by fluorescence-activated cell sorting. Haustoria were isolated as described in the *Methods* section, and fungal particles were stained with WGA-Alexa Fluor 488 (green fluorescence). **a** A CLSM image of a haustorial homogenate before cell sorting showing a haustorial cell (green) and many particles and cell debris including chloroplasts (red). **b** Counts versus log green fluorescence of particles from haustorial homogenate. The green population (R1) represents fungal particles stained with WGA-Alexa Fluor 488. **c** Log green fluorescence versus log forward scatter. The red population (R2) indicates selected cells corresponding to haustoria. Events shown as green dots (e.g., hyphal debris or spores) and black (e.g., chloroplasts or plant cell wall debris) were rejected. **d** A CLSM image showing purified haustoria after fluorescence-activated cell sorting. Bars, 25 μm

To obtain preparations of total RNA from haustorial cells of the highest quality possible, a method involving the separation of haustoria by flow cytometry was conceived. The homogenate resulting from the filtration step after the homogenization of zucchini cotyledons highly infected with *P. xanthii* was stained with wheat germ agglutinin (WGA)-Alexa Fluor 488 to label haustorial cells with a green fluorescent dye (Fig. 1a) and transferred to the cell sorter. The R1 population corresponds to fungal structures stained with WGA-Alexa Fluor 488 (Fig. 1b). The R2 population corresponding to haustorial cells was clearly identified and separated from larger or smaller particles with green fluorescence corresponding to *P. xanthii* conidia or hyphal debris, respectively (Fig. 1c). Only those particles combining high green fluorescence (R1) and the size properties of forward scatter equivalent to the size of *P. xanthii* haustorium (R2) were collected (Fig. 1d). Prior to fluorescence-activated cell sorting, only 0.02% of the particles present in the homogenate were haustoria while the rest of the homogenate corresponded mainly to chloroplasts and, to a lesser extent, to spores and hyphal debris (Fig. 1a). After separation, it was possible to obtain highly enriched haustorial preparations with a purity of 98.9% that were virtually free of visible contaminants (Fig. 1d). These samples were then subjected to RNA isolation.

### cDNA library construction, sequencing, de novo assembly and annotation of *P. xanthii* transcriptomes

From approximately 1000 haustoria purified by flow cytometry, it was possible to isolate between 5 and 20 ng of total RNA. The quality of all RNA extractions was checked in an Agilent 2100 bioanalyser, and all RNA extractions were of a medium-low quality, obtaining RIN (RNA integrity number) values between 4 and 6.4. The best RNA extraction was used to construct the cDNA library based in a combination of oligo dT and random primers, and the Illumina library, that was subsequently sequenced on an Illumina NextSeq 550 system, yielding a total of 531,447,575 paired reads. These data are available at NCBI (BioProject PRJNA393391). These reads, along with the 975,070 raw reads previously obtained from a Roche 454 system from hyphal and conidial cDNA, which was generated by oligo dT [25], were used to generate the *P. xanthii* haustorial and epiphytic transcriptomes using the TransFlow framework [27]. The Module 1 performs the pre-processing of raw Illumina reads (haustorial reads in this study) and their assembly. The Module 2 does the same but with the 454 Roche reads (epiphytic reads in this study). The Module 1 yielded 140,862,905 Illumina pre-processed reads and the Module 2 yielded 687,517 Roche 454 pre-processed reads. Only 26.5% of haustorial reads were selected after the pre-processing task, while 70.5% of epiphytic reads were selected after the same task. Furthermore, with these reads, both modules generated 30 haustorial assemblies and 3 epiphytic assemblies. The top five of haustorial assemblies and all the epiphytic assemblies are shown in (Additional file 1: Table S1). The haustorial assemblies were more distant from the reference transcriptomes than the epiphytic transcriptomes were, that could be the result of the different length of Illumina versus 454 sequence reads and the lower quality of the haustorial RNA compared with the quality of the epiphytic RNA. Finally, the best haustorial transcriptome (scOases_cat_cd_rcMin2), a scaffolded assembly from the transcriptome assembler Oases, and the best epiphytic transcriptome (ctMIRA_ctEulK29_rcCAP3) obtained after MIRA4 and EULER-SR reconciliation of primary assemblies by CAP3 (Additional file 1: Table S1), were selected to perform all the studies in this work. These transcriptomes were selected because they were the closest to the reference transcriptomes, that is, those that obtained the best parameters in comparison with the *Candida albicans* (SRR2005826) and *Neurospora crassa* (SRR100067) reference transcriptomes used for the comparative evaluation of the Module 4 of TransFlow. Both transcriptomes were annotated with Full-LengtherNext (Additional file 2; Table S2). The summary of the annotation results is shown in Table 1.

**Table 1:** Summary of unigenes and ncRNAs obtained after Full-LengtherNext annotation

Unigenes/ncRNA	Haustorial	Epiphytic
Complete unigenes	507	5217
Incomplete unigenes	8596	7745
C-terminal	983	2076
N- terminal	2189	3581
Internal	5424	2088
ncRNAs	5516	74

The poor quality of the haustorial RNA resulted in a higher number of incomplete unigenes compared to those from the epiphytic RNA (8596 haustorial vs 7745 epiphytic incomplete unigenes) and a considerably lower number of haustorial complete unigenes compared to those from the epiphytic RNA (507 haustorial vs 5217 epiphytic complete unigenes). Nevertheless, the number of predicted non-coding RNAs (ncRNAs) was much higher in the haustorial transcriptome than that in the epiphytic transcriptome (5516 haustorial vs 74 epiphytic ncRNAs).

### The analysis of the *P. xanthii* haustorial transcriptome reveals important and specific functions of the haustorium

**Fig. 2:**
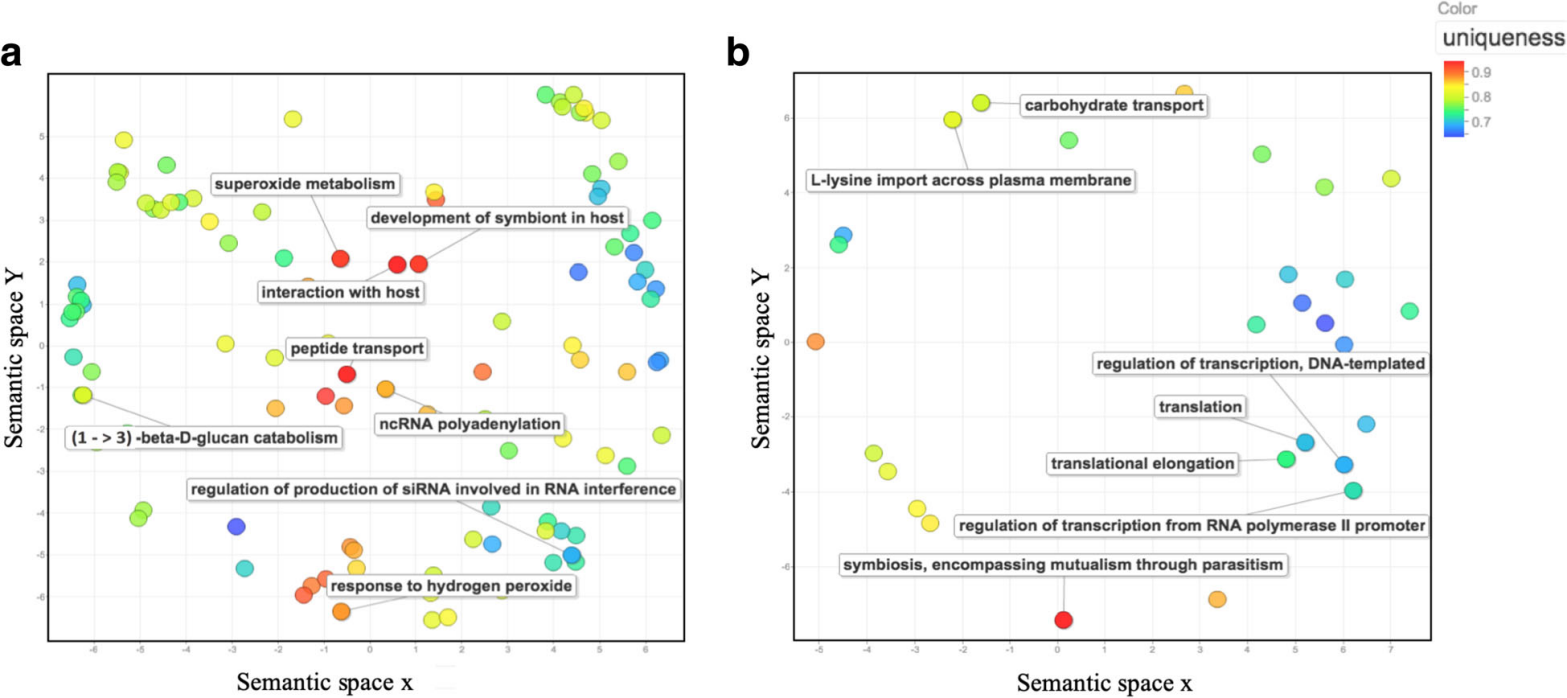
Visual representation of GO terms related to biological processes identified in the *Podosphaera xanthii* haustorial transcriptome. The server REVIGO, which condenses the GO description by removing redundant terms, with medium (0.7) allowed similarity parameter was used for this representation. The non-redundant GO terms were clustered in a two dimensional space according to semantic similarities to other GO terms. The colour of the bubble represents the “uniqueness” value of each GO term and indicates whether it is an outlier when compared semantically with the entire list. **a** Specific non-redundant haustorial GO terms. **b** Non-redundant GO terms related to biological processes of the top 50 most highly expressed haustorial genes

GO terms, retrieved from the Full-LengtherNext annotation of epiphytic and haustorial transcriptomes (Additional file 2; Table S2) were compared by Venn diagram and those that were present only in the haustorial transcriptome (Additional file 2; Table S3), were visually represented by REVIGO (Fig. 2). GO terms related to biological processes such as “development of symbiont in host” (GO:0044114) or “interaction with host” (GO:0051701), reflected the intimate relationship between haustoria and plant epidermal cells. Other GO terms were related to protection against oxidative stress, for example, “superoxide metabolism” (GO:0006801) and “response to hydrogen peroxide” (GO:0042542). GO terms such as “peptide transport” (GO:0015833) and “1,3-β-D-glucan metabolism” (GO:0006076) indicated functions related to peptide import or cell wall modification. Furthermore, other GO terms were related to the regulation of gene expression such as “regulation of production of siRNA involved in RNA interference” (GO:0090065) and “ncRNA polyadenylation” (GO:0043629). The remaining GO terms obtained after REVIGO processing that corresponded to genes exclusively expressed in haustoria are shown in (Additional file 2: Table S4).

Moreover, the top 50 most highly expressed haustorial unigenes and their associated biological processes were calculated (Additional file 2: Table S5). A list of the first 25 of them is shown in Table 2. In addition to genes involved in basic cell functions, it is interesting to note that 10 out of the top 25 most highly expressed unigenes are genes without a known function and one gene coding for an effector-like protein. GO terms related to biological processes associated with these top 50 most highly expressed haustorial unigenes are listed in (Additional file 2: Table S6). As expected, GO terms with the highest representation among the top 50 most highly expressed unigenes were “transcription” (GO:0006355, GO:0006357) and “translation” (GO:0006412, GO0006414). In addition, other GO terms that stood out were “symbiosis, encompassing mutualism through parasitism” (GO:0044403) or those related to the acquisition of nutrients such as “carbohydrate transport” (GO:0008643) and “L-lysine import across plasma” (GO:0097639).

**Table 2:** Annotation of top 25 expressed unigenes in haustoria

	FPKM	Unigene	Length (bp)	Subject_id	Description	E-value	Identity
1	3683.070	Pxanthii_hau_15484	1157	J4H557	Uncharacterized protein *Fibroporia radiculosa.*	0	65.57%
2	1119.820	Pxanthii_hau_27272	1495	P15563	Uncharacterized protein. *Podospora anserina.*	1.00E-78	47.12%
3	822.621	Pxanthii_hau_27283	1689	G3KEX6	Cytochrome c oxidase subunit 1 *Marssonina brunnea* f. sp. *multigermtubi.*	7.00E-32	81.25%
4	609.076	Pxanthii_hau_0000366852	192	A0A060QRJ0	Unassigned protein. *Fusarium culmorum* CS7071.	2.00E-05	58.33%
5	182.589	Pxanthii_hau_27316	2466	H2ET44	Uncharacterized protein. *Candida parapsilosis.*	0	47%
6	122.829	Pxanthii_hau_16140_split_1	902	P15994	ATP synthase subunit a. *Podospora anserina.*	5.00E-43	75.42%
7	117.173	Pxanthii_hau_15622	1462	N1JEF7	Drmip-Hesp domain-containing protein. *Blumeria graminis* f. sp. *hordei* DH14.	1.00E-36	56.1%
8	80.024	Pxanthii_hau_14485	477	G2YP16	Uncharacterized protein. *Botryotinia fuckeliana.*	2.00E-15	85%
9	53.860	Pxanthii_hau_27259	1389	A0A140IMW4	Laglidag endonuclease. *Pyronema omphalodes*	3.00E-109	59.88%
10	44.516	Pxanthii_hau_13721	896	C5K441	Laglidadg endonuclease. *Ajellomyces dermatitidis*	0.044	52.63%
11	43.528	Pxanthii_hau_15600	1334	P0CY45	NADH-ubiquinone oxidoreductase. *Neurospora crassa*	3.00E-40	50%
12	40.277	Pxanthii_hau_25818	257	Q08656	ATP synthase protein. *Neurospora crassa*	1.00E-18	72.92%
13	37.807	Pxanthii_hau_27,213	1001	C3VER8	Putative uncharacterized protein. *Cadophora finlandica.*	9.00E-61	59.49%
14	32.385	Pxanthii_hau_15585	1295	A0A0B1P6R5	Uncharacterized protein. *Uncinula necator*.	0.044	90%
15	30.763	Pxanthii_hau_10927	1975	L8B996	Uncharacterized protein. *Phlebia radiata*.	0.001	47.92%
16	29.426	Pxanthii_hau_27286	1766	H6D5E8	Laglidadg endonuclease *Nectria haematococca*	1.00E-139	54.63%
17	27.922	Pxanthii_hau_12650	366	K1XD01	Acyl CoA binding protein. *Marssonina brunnea* f. sp. *multigermtubi*	3.00E-28	65.66%
18	25.013	Pxanthii_hau_8297	2994	M1F6T6	Endonuclease *Ceratocystis cacaofunesta*	0.006	45.28%
19	24.719	Pxanthii_hau_15,673	1872	N1JQK7	Uncharacterized protein. *Blumeria graminis* f. sp. *hordei* DH14.	0	53.81%
20	24.349	Pxanthii_hau_27058	599	N0A396	Uncharacterized protein. *Rhizoctonia solani.*	0.25	58.62%
21	18.995	Pxanthii_hau_15091	1056	Q7RZA5	S-methyl-5′-thioadenosine phosphorylase. *Neurospora crassa.*	2.00E-156	66.88%
22	17.801	Pxanthii_hau_15,569	1893	A0A061HDU5	Repressible acid phosphatase *Blumeria graminis* f. sp. *tritici* 96,224.	0	61.04%
23	17.560	Pxanthii_hau_689	2258	N1JHJ7	CELP0015 Effector like protein *Blumeria graminis* f. sp. *hordei* DH14	0	65.35%
24	13.644	Pxanthii_hau_27311	2381	P00908	Multifunctional tryptophan biosynthesis protein *Neurospora crassa*	0	62.57%
25	13.521	Pxanthii_hau_27288	1835	A0A0B1PB18	Putative repressible acid phosphatase. *Uncinula necator.*	0	60.34%

**Fig. 3:**
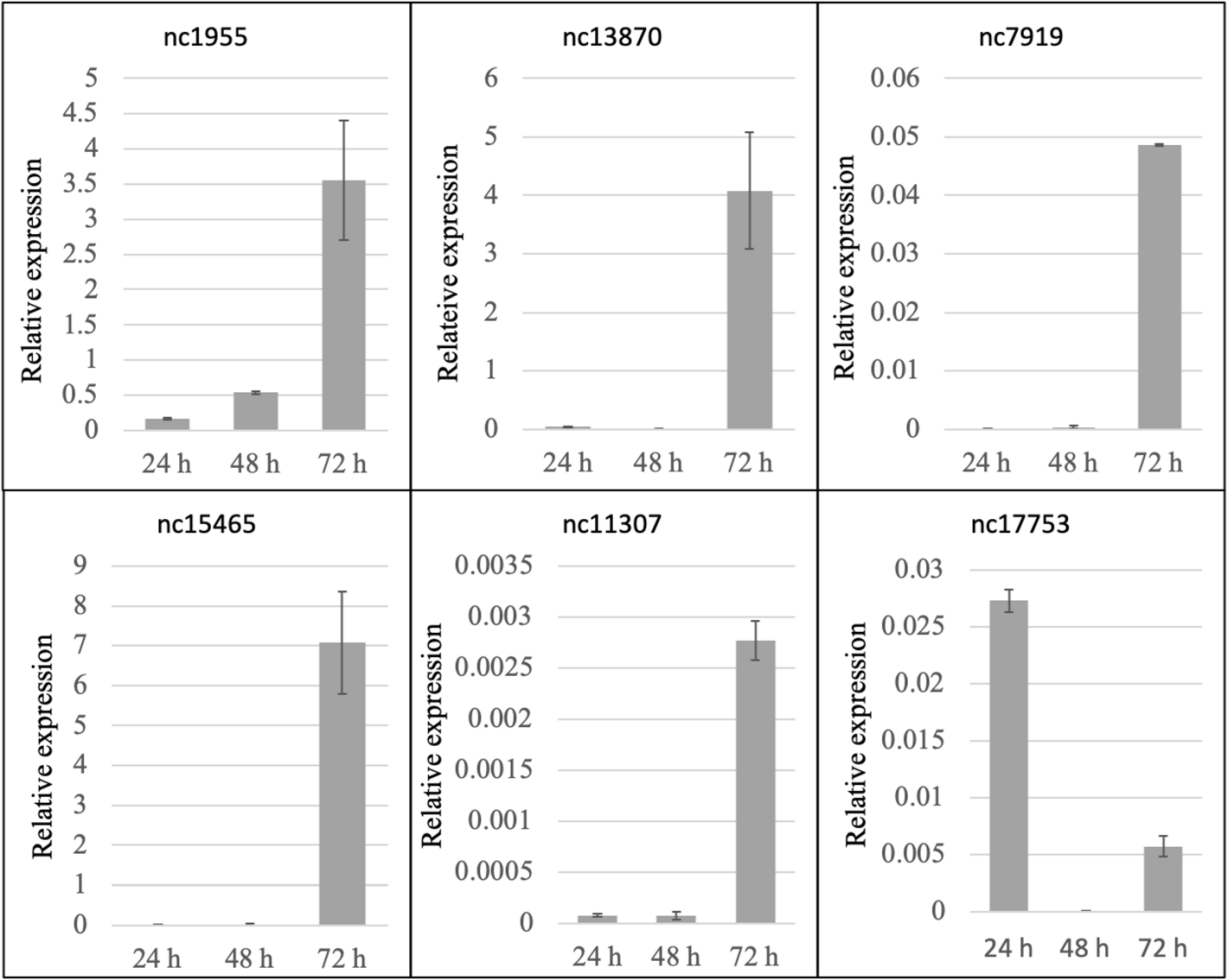
Analysis of the relative expression of *Podosphaera xanthii* selected ncRNAs during the first stages of infection. Total RNA was isolated from zucchini cotyledons inoculated with *P. xanthii* at different time points (24, 48 and 72 h post-inoculation), and the relative expression of ncRNAs was analysed by qRT-PCR. Transcript abundance was normalized to the transcription of the endogenous control elongation factor-1 gene *PxEF1* (MK249653). The data indicate the average values of three independent experiments with three experimental replicates. The error bars indicate the standard error

In addition, the trimmed reads of the haustorial transcriptome were also aligned against all ncRNAs and only those with 10 or more mapping reads were selected in order to avoid possible artefacts. Among the 5516 predicted ncRNAs by Full-LenghterNext annotation only 2143 showed 10 or more mapping reads and, hence, were selected for further analysis. The top 50 most highly expressed ncRNAs of this group of 2143 predicted ncRNAs were calculated and annotated using the RNAcentral database (Additional file 2: Table S7). The annotation of these top 50 expressed ncRNAs only showed a few putative functions (Table 3). Among them, two ncRNAs (nc15465 and nc13870) were present in other powdery mildew fungi, *Erysiphe uncinuloides* and *Blumeria graminis*, respectively, whereas most of them were annotated with partial sequences of lncRNA from *Homo sapiens*. Moreover, the RNase function was quite represented among top 50 highly expressed ncRNAs. The expression pattern of 6 of these ncRNAs during the first stages of *P. xanthii* infection was very similar (Fig. 3), with a very low expression at 24 and 48 h post-inoculation and a high increase in transcript levels at 72 h post-inoculation. The only exception was nc17753, which was putatively annotated as *Homo sapiens* lncRNA, which showed a wave-like expression pattern with a high expression at 24 h post-inoculation, followed by a decrease at 48 hpi and a high increase at 72 hpi. Three of the analysed ncRNAs (nc1955, nc13870 and nc15465) showed a relative high expression at 72 hpi.

**Table 3:** Annotation of selected haustorial ncRNAs

Sequence ID	Lenght (bp)	RNAcentral ID	Description^a^	E-value	Identity	Target coverage
Pxanthii_hau_17753	290	URS00008C38E6	*Homo sapiens* lncRNA	3.80E-65	74.3%	43%
Pxanthii_hau_15465	1021	URS0000DECADB	*Erysiphe uncinuloides* miscRNA	1.50E-37	65.9%	99.7%
Pxanthii_hau_7919	238	URS0000A7736E	*Arabidopsis thaliana* signal recognition particle (SRP)	3.30E-17	66.4%	77.9%
Pxanthii_hau_13870	383	URS0000BF022A	*Blumeria graminis* f. sp. *triciti* RNase P RNA	1.30E-45	69.5%	99.2%
Pxanthii_hau_1955	253	–	–		–	–
Pxanthii_hau_11307	257	URS000071105	*Prunus persica* RNase MRP RNA	5.40E-23	66.5%	94.6%

### Ten secreted proteins are predominantly expressed in the haustorium

**Fig. 4:**
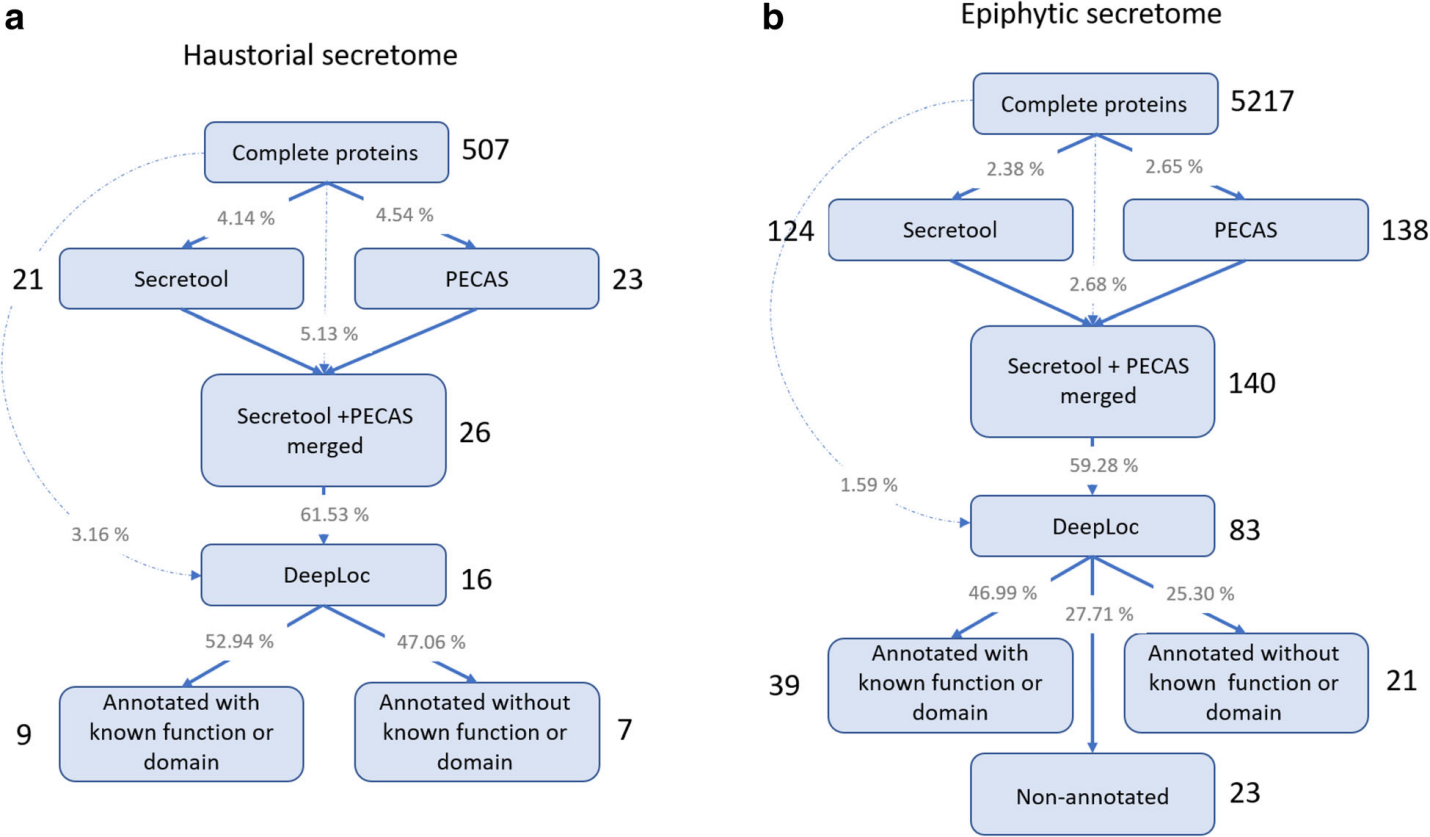
Workflow used to define the *Podosphaera xanthii* epiphytic and haustorial secretomes. The software used is indicated in light blue boxes, and the number of proteins selected after each software analysis is indicated with a number outside the corresponding box. The grey numbers show the percentage of secreted proteins obtained in each step of the secretome prediction workflow. **a** The *P. xanthii* haustorial secretome is predicted to be composed of 16 proteins, among which 9 were annotated with known function or domain and 7 were annotated without known function or domain. **b** The *P. xanthii* epiphytic secretome is predicted to be composed of 83 proteins, among which 39 were annotated with known function or domain, 21 were annotated without known function or domain and are conserved in fungi and 23 were non-annotated

**Fig. 5:**
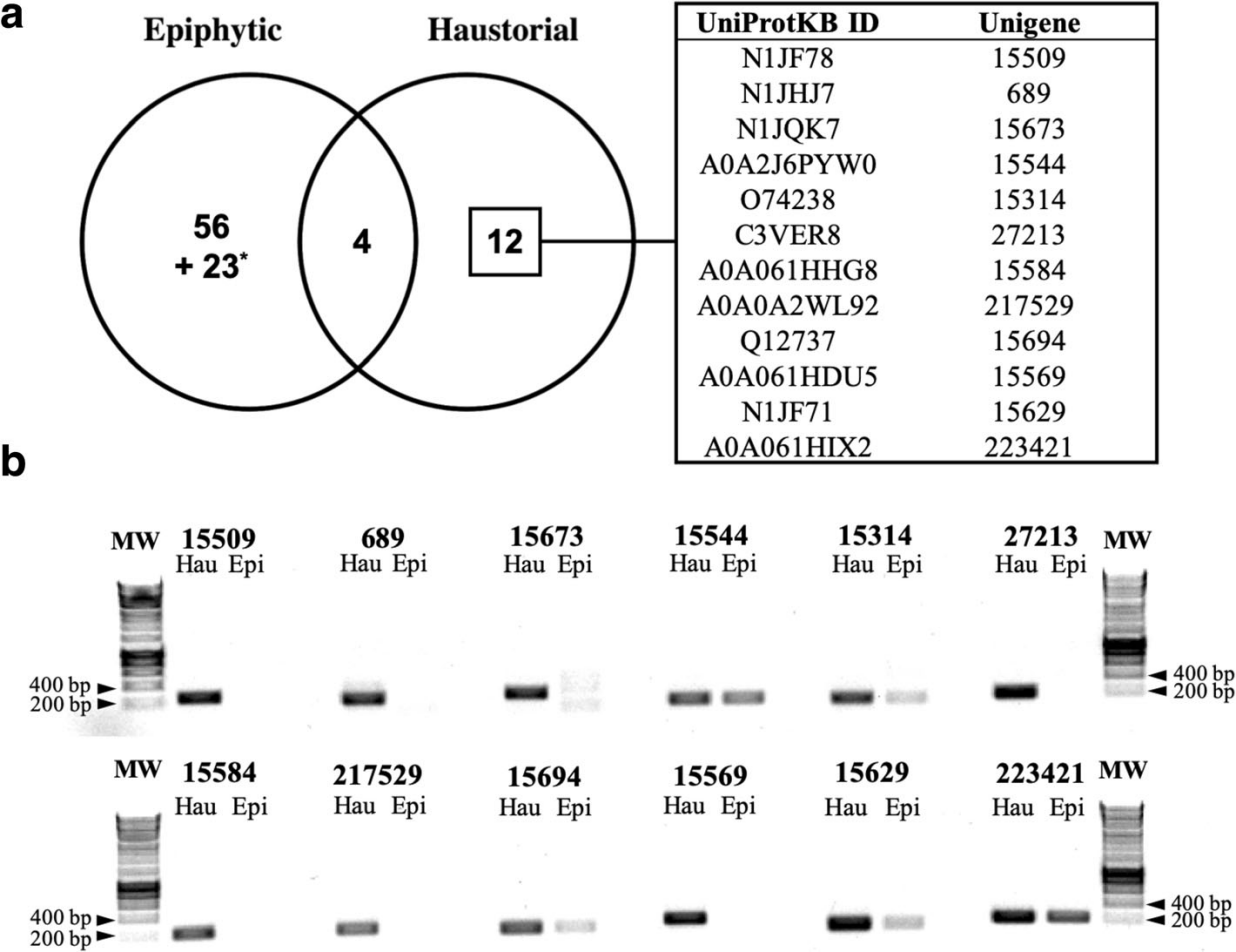
Identification and validation of haustorium-specific secreted proteins. **a** Venn diagram showing the identification of *Podosphaera xanthii* haustorium-specific secreted proteins. The comparison was carried out using the UniProtKB protein identifiers of complete and annotated secreted proteins deduced from the revised epiphytic and the haustorial transcriptomes of *P. xanthii*. The box on the right shows the UniProtKB protein ID and unigene name of the *P. xanthii* haustorium-specific proteins. The asterisk denotes the set of non-annotated proteins detected in the epiphytic secretome. **b** Detection of transcripts of *P. xanthii* haustorium-specific secreted proteins in haustorial (Hau) and epiphytic (Epi) structures. Isolation of epiphytic structures and haustorial cells, extraction of RNA and cDNA synthesis were performed as described in Methods. Detection of transcripts was carried out by PCR analysis using the specific primers listed in (Additional file 1: Table S11). The picture shows a representative image of an agarose gel with the results obtained after 3 different PCR experiments with three different RNA samples MW, molecular weight marker HyperLadder 1Kb (Bioline, London, UK)

The free software tools Secretool and PECAS were used to obtain the predicted haustorial and epiphytic secretomes from complete proteins. These initial secretomes were further processed by DeepLoc to obtain a more accurate prediction of secreted proteins (Fig. 4). After Secretool and PECAS secretome processing, 26 haustorial and 140 epiphytic proteins were selected; however, only 61.5% of the haustorial proteins and 59.3% of the epiphytic proteins were validated as secreted proteins by DeepLoc (Additional file 2: Table S8). In this way, a total of 16 haustorial and 83 epiphytic secreted proteins were obtained (Additional file 2: Table S9), corresponding to 3.16 and 1.59% of the total of haustorial and epiphytic complete proteins, respectively. Additionally, UniProtKB identifiers were retrieved from the Full-LengtherNext annotation results and used to compare the haustorial and epiphytic secreted proteins (Fig. 5a). Among the 16 transcripts encoding proteins predicted to be secreted, 12 of them were represented exclusively in the haustorium according to UniProtKB identifiers (Fig. 5a). To test the haustorial specificity of these 12 transcripts, semiquantitative RT-PCR analysis was conducted using cDNA obtained from epiphytic mycelia/conidia and isolated haustoria, and specific primers for each gene (Fig. 5b). Results of this experiment showed that 10 of them were exclusively (15,509, 689, 15,673, 27,213, 15,584, 217,529, 15,559) or mainly (15,314, 15,694, 15,629) expressed in haustoria and, therefore, they were denominated gene encoding haustorium-specific secreted proteins (Table 4). From this 10 genes, 7 of them were annotated without known function or motif. These haustorium-specific genes encoding secreted proteins without functional annotation were then designated *Podosphaera* Haustorial Effector Candidates (PHECs).

**Table 4:** Annotation of *P. xanthii* genes encoding haustorium-specific secreted proteins

Sequence ID	PHEC name	Protein^a^	Subject ID	Description	E-value	Identity
Pxanthii_hau_689	PHEC689	457	N1JHJ7	CELP0015 Effector like protein. *Blumeria graminis* f. sp. *horde*i DH14	0.0	65.35%
Pxanthii_hau_15,509	PHEC15509	339	N1JF78	Uncharacterized protein *Blumeria graminis* f. sp. *horde*i DH14	1.0e-91	47.55%
Pxanthii_hau_15,314	–	135	O74238	Protein SnodProt1. *Phaeosphaeria nodorum*	4.0e-44	51.47%
Pxanthii_hau_15,629	–	401	N1JF71	Thioredoxin reductase. *Blumeria graminis* f. sp. *horde*i DH14	3.0e-137	58.07%
Pxanthii_hau_15,569	–	538	A0A061HDU5	Repressible acid phosphatase. *Blumeria graminis* f. sp. *horde*i 96,224.	0.0	61.04%
Pxanthii_hau_15,584	PHEC15584	249	A0A061HHG8	Uncharacterized protein. *Blumeria graminis* f. sp. *horde*i 96,224.	1.0e-43	49.34%
Pxanthii_hau_15,673	PHEC15673	461	N1JQK7	Uncharacterized protein. *Blumeria graminis* f. sp. *horde*i DH14	0.0	53.81%
Pxanthii_hau_15,694	–	602	Q12737	Bilirubin oxidase. *Myrothecium verrucaria.*	4.0e-145	47.51%
Pxanthii_hau_27,213	PHEC27213	209	C3VER8	Putative uncharacterized protein. *Cadophora finlandica.*	6.00e-71	53%
Pxanthii_hau_0000217529	PHEC217529	86	A0A0A2WL92	Uncharacterized protein. *Beauveria bassiana* D1–5	1.00E-51	99%

^a^ Number of amino acids in the unprocessed form (with signal peptide)

### Protein modelling and protein-ligand predictions reveal putative functions for some PHECs

**Fig. 6:**
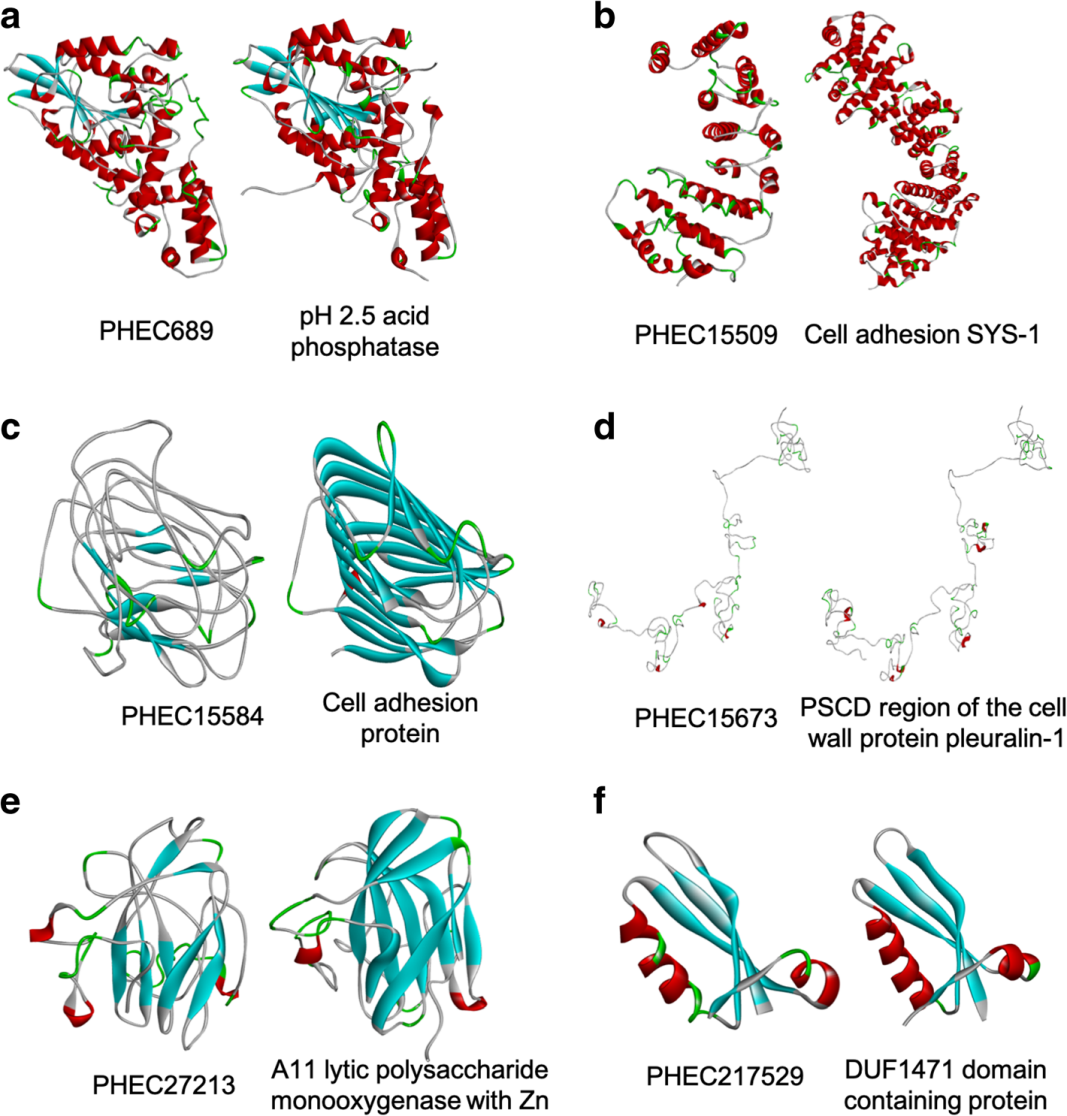
Predicted three-dimensional (3D) models of *Podosphaera xanthii* haustorium-specific effector candidates (PHECs) and their best structural analogues constructed using the I-TASSER server. **a** PHEC689 and an acid phosphatase from *Aspergillus niger* (PDB code 1QXF). **b** PHEC15509 and the cell adhesion protein SYS-1 from *Caenorhabditis elegans* (PDB code 3C2H). **c** PHEC15584 and a cell adhesion protein from *Clostridium sporogenes* (PDB code 4QRK). **d** PHEC15673 and the PSCD region of the cell wall protein pleuralin-1 from *Cylindrotheca fusiformis* (PDB code 2NBI). **e** PHEC27213 and the A11 lytic polysaccharide monooxygenase from *Aspergillus oryzae* (PDB code 4MAH). **f** PHEC217529 and the DUF1471 domain containing protein from *Salmonella typhimurium* (PDB code 2M2J)

To decipher the biological functions of these PHECs without known function, protein models were obtained for each PHEC using the I-TASSER server and the amino acid sequence of the mature protein (without a signal peptide). Additionally, a set of prediction tools (Phyre2, CATH/Gene3D and Motif Scan) was used to obtain more information related to the putative functions or domains of PHECs. The resulting I-TASSER models are shown in Fig. 6, and the main features retrieved from all software used are listed in Table 5.

**Table 5:** Principal features of PHECs obtained after structure modelling

Protein model	Score values^a^		Structural analogs^b^		Predicted features	
	C score	TM score	PDB file	Species	Activity^c^	Ligands^d^
PHEC689	0.98	0.901	1QFX	*Aspergillus niger*	Histidine acid phosphatase	Phosphate ion
PHEC15509	−3.70	0.733	3C2H	*Caenorhabditis elegans*	Cell adhesion protein similar to integrin	Peptide
PHEC15584	−3.69	0.749	4QRK	*Clostridium sporogenes*	Cell adhesion protein	N-acetylglucosamine
PHEC15673	−0.26	0.934	2NBI	*Cylindrotheca fusiformis*	Cell wall protein pleuralin-1 with serine-rich region	Peptide
PHEC27213	−2	0.715	4MAH	*Aspergillus oryzae*	Lytic polysaccharide monooxygenase with chitin binding domain	Zinc
PHEC217529	0.87	0.856	2M2J	*Salmonella typhimurium*	Flavin-binding protein dodecin	Nucleic acid

^a^Score values represent the reliability of each model according to I-Tasser. C-score values measure the confidence of each model and are in the range of −5 to 2, where a higher value means a model with a higher confidence. TM-score values measure the structural similarity between two protein models. TM-score is in the range of 0 to 1, being 1 a perfect match between the models

^b^Protein structurally close to the PHEC model according to I-TASSER prediction. PDB = Protein Data Bank

^c^Putative activity based on the results obtained from software tools I-TASSER, Phyre2, CATH/3D gene and Motif Scan

^d^Ligand prediction according to COACH software

The PHEC689 model exhibited significant structural analogy with an acid phosphatase from *Aspergillus niger* (PDB code 1QFX) (Fig. 6a; Table 5) and a phosphate ion as protein ligand (Table 5), suggesting that PHEC689 could be able to release phosphate groups from different plant molecules. The PHEC15509 model showed structural analogy with the cell adhesion protein SYS-1 from *Caenorhabditis elegans* (PDB code 3C2H) as well as a putative interaction with peptides (Fig. 6b; Table 5). For its part, CATH/GENE 3D and Phyre2 indicated analogy with an integrin protein, suggesting that PHEC15509 could act as an integrin involved in cell adhesion by binding to other proteins. The resulting PHEC15584 model showed structural analogy with a cell adhesion protein from *Clostridium sporogenes* (PDB code 4QRK) and N-acetylglucosamine was predicted as a ligand (Fig. 6c; Table 5), suggesting that PHEC15584 could act as a cell adhesion protein that interacts with N-acetylglucosamine residues. In the case of PHEC15673, the resulting model presented significant structural analogy with the PSCD (proline, serine, cysteine and aspartate) domain of cell wall pleuralin-1 from diatom *Cylindrotheca fusiformis* (PDB code 2NBI) as well as a serine-rich region and a possible interaction with peptides (Fig. 6d; Table 5), suggesting a putative function related to adhesion. The PHEC27213 model showed high structural analogy with an A11 lytic polysaccharide monooxygenase from *Aspergillus oryzae* (PDB code 4MAH) and a chitin binding domain (Fig. 6e; Table 5)*,* suggesting, that PHEC27213 could act as a chitin-active lytic polysaccharide monooxygenase. Finally, the resulting PHEC217529 model showed significant structural analogy with a DUF1471 domain-containing protein from *Salmonella typhimurium* (PDB code 2M2J) (Fig. 6f; Table 5) whereas the predicted features retrieved from CATH/Gene3D and Phyre2 analyses indicated analogy with a flavin-binding protein dodicin.

### Expression analysis of *P. xanthii* haustorium-specific secreted proteins

**Fig. 7:**
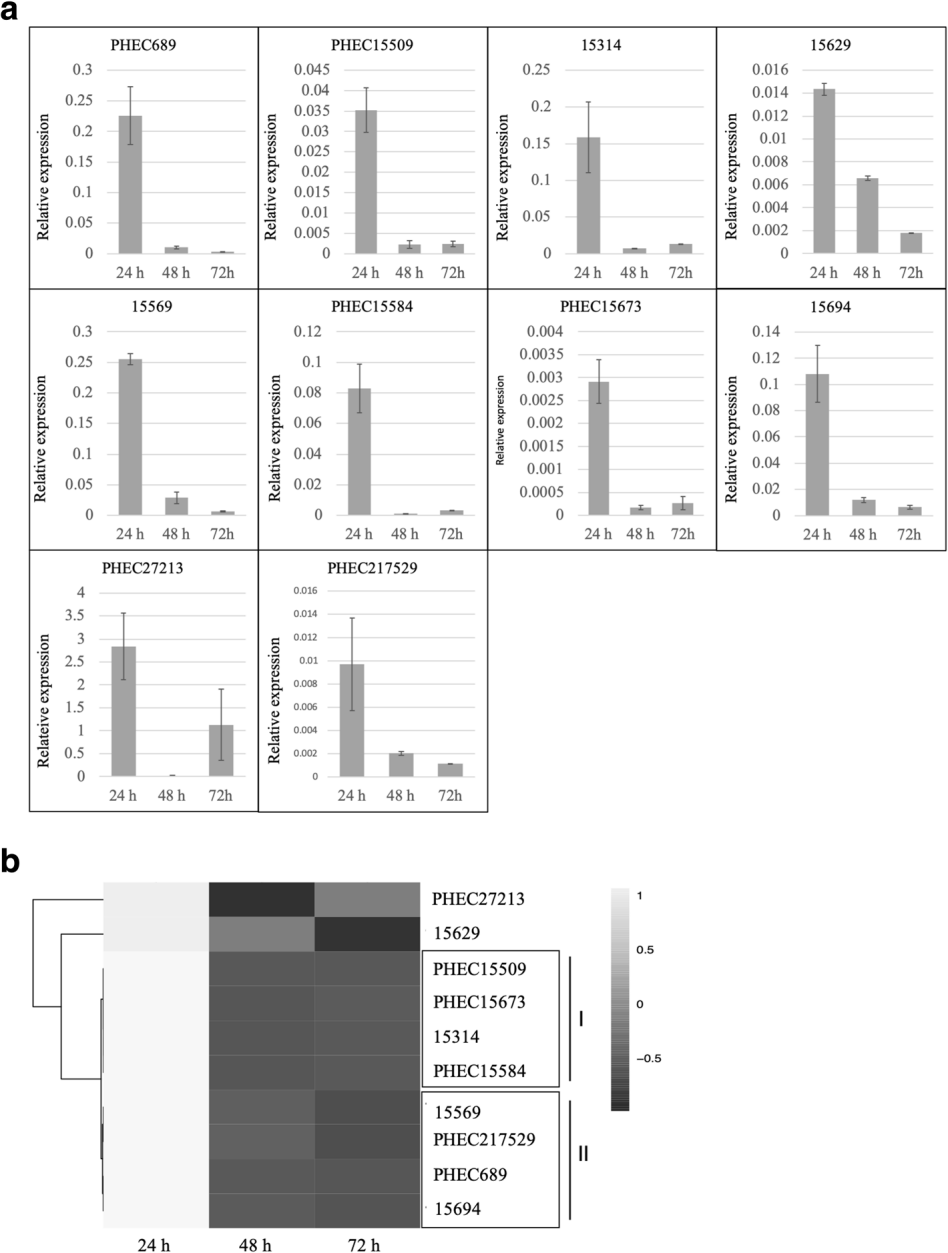
Analysis of the relative expression of *Podosphaera xanthii* genes coding for haustorium-specific secreted proteins during the first steps of infection. **a** Relative expression of genes analysed by quantitative reverse transcription–polymerase chain reaction (qRT-PCR). Total RNA was isolated from zucchini cotyledons inoculated with *P. xanthii* at different time points (24, 48 and 72 h post-inoculation), and the relative expression of genes was analysed by qRT-PCR. Transcript abundance was normalized to the transcription of the endogenous control elongation factor-1 gene *PxEF1* (MK249653). The data shown represent average values of three experimental replicates from three independent experiments, with error bars showing the standard error. **b** Hierarchical clustering of the relative expression of genes coding for haustorium-specific secreted proteins at different time points after inoculation (24, 48 and 72 h) performed by ClustVis. Rows are clustered using distance and average linkage. Changes in gene expression are displayed from white (higher expression) to black (lower expression)

The expression patterns of the ten *P. xanthii* genes coding for haustorium-specific secreted proteins were analysed during the first stages of pathogenesis (Fig. 7a). In addition, these genes were grouped by hierarchical clustering according to their expression levels (Fig. 7b)*.* All showed a similar expression pattern with high levels of expression at 24 h followed by a sharp decrease in expression at 48 h, whereas at 72 h, the expression increased or decreased in comparison to 48 h. The genes that exhibited a second increase in expression at 72 h were grouped into cluster I. For its part, the genes that showed a slight decrease in expression at 72 h were grouped into cluster II. In the cases of *15,629* and *PHEC27213*, they were not grouped in any cluster, since the decrease in expression of *15,629* was attenuated in time without the abrupt decrease in expression that occurs in most genes, and the increase in expression of *PHEC27213* at 72 h was sharper than in other genes. The genes with the highest relative expression were *PHEC27213*, *15,569* and *PHEC689*, which, interestingly, were found to be among the top 25 most highly expressed haustorial genes (Table 2).

## Discussion

Powdery mildew is a devastating disease in cucurbits. *P. xanthii* is its main causal agent and is responsible for significant yield losses in many cucurbit crops worldwide [4]. The haustorium, which is a specialized structure for parasitism in powdery mildew fungi, plays a pivotal role in biotrophy and pathogenesis [7] and allows an intimate interaction between the fungus and the plant cells. Therefore, it is an essential element in the establishment of powdery mildew disease. However, despite its biological importance, the only study on the *P. xanthii* haustorium is a microscopic analysis of its structure [5]. In a previous study, the role of several effector candidates secreted by *P. xanthii* during epiphytic growth in powdery mildew pathogenesis was demonstrated [6]. In this work, we sought to decipher the molecular bases for the functions of the *P. xanthii* haustorium and find those effector candidates expressed specifically in this structure. This is, in our opinion, valuable information to help unveil the unknown aspects of the biotrophy and pathogenesis of powdery mildew fungi. For this purpose, a method to isolate haustoria free of contaminants was developed that was used together with the de novo assembly of the haustorial transcriptome, the corresponding secretome prediction and the comparison between the haustorial transcriptome and the epiphytic transcriptome.

Transcriptomic studies have been used to obtain relevant information about gene expression profiles in powdery mildew fungi [21, 22]; thus, they have allowed for more accurate effector prediction than the prediction obtained from powdery mildew genomes [20]. However, the difficult task of isolating the haustoria is reflected in the few haustorial transcriptomic studies available to date [23, 24]. Due to the high presence of plant contaminants in the haustorial preparations obtained either by isopycnic Percoll centrifugation [23, 24] or after elution from concanavalin A columns [28], we developed a method to isolate haustoria by flow cytometry that allowed us to obtain haustorial cells virtually free of visible contaminants. However, all these techniques require considerable time and, therefore, can alter the gene expression status of haustorial genes and/or damage the RNA quality [23] since the most unstable mRNAs have a half-life of only 5 min and could degrade, as previously described in yeast [29]. For this reason, the isolation of low-quality RNA is expected, although due to the difficulties in isolation and the small amount of molecular information about the haustorium currently available, the risk of the loss of sequence information is acceptable.

The sequencing of low-quality RNA is not recommended; however, it has been shown that useful data can be collected using highly degraded RNA [30]. A decrease in RIN values generally results in an increase in 3′ bias; however, RIN values between 4 and 6 correspond with 62.2 and 58.1% 3′ bias, respectively, versus the 50% 3′ bias expected for an RIN of 10 [31]. Moreover, the amplification using oligo (dT) and random primers carried out by the Ovation RNA-Seq System V2 decreased this bias due to the simultaneous amplification across the whole transcriptome and at the 3′ end, yielding an accurate and uniform transcriptome representation [32]. In addition, the customized InDA-C primers used by the Ovation RNA-Seq System V2 to deplete ribosomal sequences considerably reduced the rRNA contamination in the final reads [33]. All this, together with the yield from Illumina NextSeq 550 sequencing (531,447,575 sequence reads), became the best option to obtain as much sequence information as possible from the low-quality haustorial RNA. Despite this, only 26.5% of the haustorial reads were useful after pre-processing versus 70.5% of the epiphytic reads. This can be explained by the presence of rRNA and tRNA sequences not depleted by the InDA-C primers that, therefore, produced a large number of filtered out reads from the haustorial RNA. This, together with the lower number of complete coding unigenes obtained from the haustorial transcriptome compared with those from the epiphytic transcriptome, confirmed the difficulties associated with the isolation of haustorial RNA.

Although the haustorial transcriptome of *P. xanthii* is probably largely incomplete, its characterization and comparison with the epiphytic transcriptome have allowed us to identify certain biological processes specific to the haustorium such as “development of symbiont in host” and “interaction with host” that show the intimate relationship between the haustorium and plant cells. Consistent with this, the transcription factor CPh2 (Pxanthii_hau_15645), which is related to the positive regulation of filamentous growth, virulence and invasiveness in *Candida albicans* [34, 35], was found in the top 50 most highly expressed genes. Other haustorial-specific processes were “superoxide metabolism” and “response to hydrogen peroxide”, which included the specific expression of genes involved in protection against oxidative stress, suggesting a key role of the haustorium in ROS scavenging as previously described in other transcriptomic and proteomic studies of powdery mildew and rust haustoria [19, 36–39]. Similarly, the process “1,3-β-D-glucan catabolism” suggests the expression of specific haustorial genes related to the modification of the fungal cell wall during development of the haustorium, although some of these genes could also be involved in the degradation of plant β-glucans such as callose, a 1,3-β-glucan present in papillae, as described in other fungal plant pathogens [40]. Nutrient uptake is a typical function of haustoria of powdery mildew and rust fungi [23, 36–38, 41, 42]. The haustorial-specific process “peptide transport” corresponded to the dipeptide transport ATP-binding protein DppF, indicating the presence of a peptide uptake system [43, 44], which plays a key role in the human bacterial pathogen *Treponema denticola* [44]*.* With this regard, other transporters such as an amino-acid permease and a MFS sugar transporter were found among the top 50 most highly expressed genes in the *P. xanthii* haustorium.

On the other hand, the considerably high number of ncRNAs detected in haustoria, together with specific haustorial biological processes identified such as “regulation of production or siRNA involved in RNA interference” and “ncRNA polyadenylation”, suggest the importance of this type of genetic regulation by the haustorium, making it tempting to speculate that an important part of these ncRNAs could be related to pathogenesis, as previously described in *Phytophthora infestan* [45]*.* Six of these ncRNAs (five of them with high coverage and identity in comparison with the annotated ncRNA and one of them without putative function), were selected among the top 50 expressed ncRNAs and subjected to further analysis. It is noteworthy the presence among these 6 putative ncRNAs of a signal recognition particle (SRP), which catalyze targeting of nascent secretory proteins, as well as a RNase P and a RNase MRP, which are involved in tRNA and rRNA processing, respectively. The high expression of these ncRNAs supports a high translation, a biological process highly represented among top 50 expressed genes. In concordance with our results, previous reports suggested that small RNAs might play a role in the plant-powdery mildew interaction [46–49].

In the originally reported epiphytic transcriptome [25], 137 secreted protein candidates were identified. In the revised version of the transcriptome obtained in this work, 140 secreted protein candidates have been proposed. Despite the slight increase in the number of these proteins, the use of DeepLoc software significantly reduced the number of predicted secreted proteins. This fact demonstrated the restrictive approach used to define the predicted secretomes that allowed us to obtain a more accurate prediction and reduced the rate of false positives, despite losing several putative secreted protein candidates. The number of haustorial and epiphytic secreted proteins predicted in comparison to the total number of complete unigenes (3.2% of the complete haustorial proteins are candidate secreted proteins versus 1.6% of the complete epiphytic proteins), suggests that the haustorium contributes to the secretion of proteins to a great extent, highlighting the contribution of this structure to the process of protein secretion [25, 50] and consequently to pathogenesis [7].

The previously obtained epiphytic transcriptome [25] has allowed us to perform a comparison with the haustorial transcriptome and select those effector candidates expressed exclusively in the haustorium. Although most of them have a non-annotated function, the reliability of I-TASSER and other software in the protein function prediction of *P. xanthii* effectors had been demonstrated in a previous study [6]. In this work, a similar in silico analysis was carried out, revealing putative functions for several haustorium-specific secreted proteins such as phosphorous acquisition, cell adhesion, cell wall degradation/modification and defence against oxidative stress.

Regarding the latter function, the detection of 15,629 and PHEC217529 transcripts, which code for a putative thioredoxin reductase and a putative flavin-binding dodecin protein, respectively, among the haustorium-specific secreted proteins emphasizes the importance of protecting the haustorium against plant oxidative stress. Thioredoxin reductase has been described as involved in defence against reactive oxygen species [51, 52] and is also necessary for cell wall integrity of *Magnaporthe oryzae* during biotrophic colonization of the host [53]*.* Similarly, protection against oxidative stress has been suggested as a function of dodecin proteins [54]. Dodecins are widely spread and found in a large variety of bacterial pathogens including plant pathogens such as *Ralstonia solanacearum*. However, their role in pathogenesis has not been identified until very recently; specifically, their role in *Mycobacterium tuberculosis* pathogenicity was identified [55].

Two putative acid phosphatases (15,569, PHEC689) were found among the effector candidates specifically expressed in the haustorium. The implication of these enzymes in pathogenesis has been described in intracellular bacteria such as *Francisella tularensis* [56, 57], the tuberculosis agent [58] and the entomopathogenic fungus *Metarhizium anisopliae* [59]. Moreover, their coordination (grouped in cluster II), high relative expression (among the top 50 most highly expressed haustorial genes) and function (phosphate acquisition) support their importance in biotrophy rather than in pathogenesis because they present an expression pattern and a molecular size different from those described for canonical powdery mildew effectors [19, 50]. Nevertheless, pathogenesis and survival in the host follow a very thin line in biotrophic biology, and sometimes it is very difficult to distinguish what is specific for pathogenesis from basic physiological functions.

Other specific haustorial proteins in *P. xanthii* seem to be canonical effectors. This is the case for 15,314, a putative homologue of SnodProt1, a virulence factor in *P. nodorum* of unknown function [60]. Within this group, we could also include 15,629, a putative bilirubin oxidase, and PHEC27213, a putative A11 lytic polysaccharide monooxygenase. These enzymes have been previously described in fungi but play a role in biomass conversion rather than a role in pathogenesis [61–63]. However, the typical effector expression pattern of PHEC27213 and the fact that it was the most highly expressed haustorial effector candidate and the thirteenth most expressed haustorial gene suggest a potentially important role in *P. xanthii* pathogenesis.

Interestingly, a third of the total specific haustorial secreted proteins correspond to cell adhesion proteins (PHEC15509, PHEC15584, PHEC15673) that present the same expression pattern (cluster I). Integrin proteins (such as putative integrin PHEC15509) have a relevant role in adhesion to host proteins in *C. albicans* [64, 65]. To date, no proteins with cell adhesion properties have been described in biotrophic fungi. Nevertheless, the high number of these proteins among all haustorium-specific secreted proteins suggests the relevance of cell adhesion proteins to the biotrophic lifestyle, perhaps as haustorial-stabilizing proteins during the development and accommodation of the haustorium inside the plant cell.

The results of this study, the third study on powdery mildew haustoria, raise an interesting question: is it possible to identify gene sets that are generally haustorium-specific? The most expressed biological processes identified in the *P. xanthii* haustorium such as transcription and translation, have been described in *G. orontii* haustorial transcriptome [23], supporting the high protein turn-over in haustoria. However, the same is not true for most haustorium-specific secreted proteins. Only two proteins with similar functions to those described in *P. xanthii*, ROS scavenging, such as a Cu/Zn superoxide dismutase, and chitin modification, such as a chitinase, were described in the *G. orontii* haustoria [23], whereas none of them were described in the haustorial transcriptome of *B. graminis* [24]. This difference can be due in part to the high number of secreted proteins without functional annotation in those transcriptomic studies. In addition, the quality of haustorial transcriptomes is often poor, so it is easy to assume that substantial information is missing. However, the fact that for most of the genes encoding haustorial-specific *P. xanthii* secreted proteins is possible to find orthologues in the genomes of other powdery mildew fungi, suggests that there is a possibility that haustorial core genes will be found in the future.

## Conclusions

**Fig. 8:**
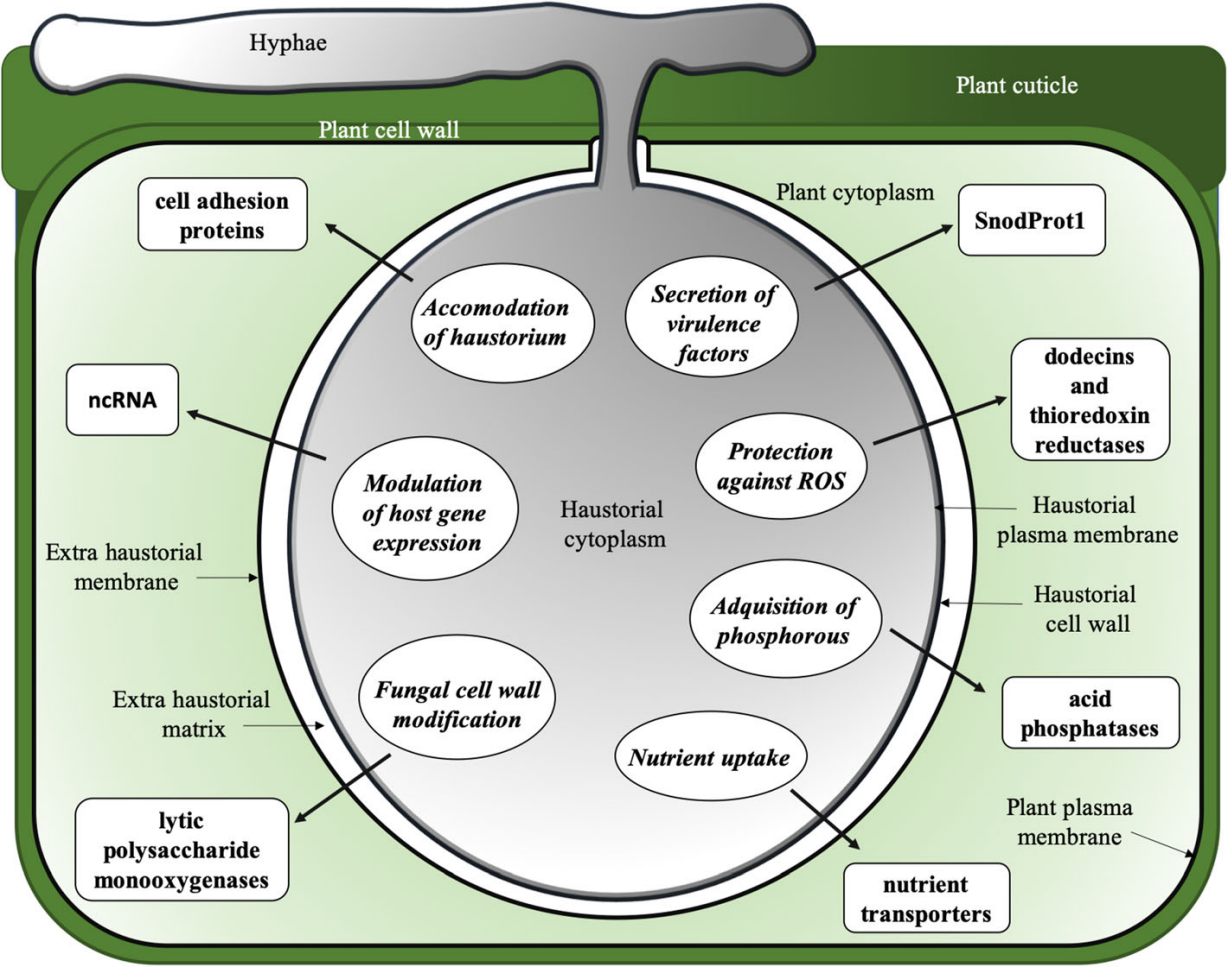
Schematic representation of the physiological processes specific to the *P. xanthii* haustorium according to the results obtained in this work

Despite the difficulties in obtaining preparations of haustoria free of contaminants and in isolating good quality RNA from these cells, the assembly of a de novo haustorial transcriptome of *P. xanthii* and its comparison with a revised version of an epiphytic transcriptome previously developed allowed us to gain new insights into the biotrophy and pathogenesis of *P. xanthii.* According to specifically expressed genes, major biological functions associated with the haustorium such as protection against reactive oxygen species and the acquisition of nutrients were supported. However, additional important functions such as the secretion of cell adhesion proteins have not been described to date. A schematic representation of the functions of the *P. xanthii* haustorium according to the results obtained in this work is shown in Fig. 8. The analysis of these functions could be a nice starting point to unravel the unknown aspects of powdery mildew biology.

## Methods

### Fungal and plant material

The *Podosphaera xanthii* isolate 2086 was routinely cultured on previously disinfected zucchini (*Cucurbita pepo* L.) cotyledons cv. Negro Belleza (Semillas Fitó, Barcelona, Spain) and maintained in vitro in Bertrand medium in 8 cm Petri dishes under a 16 h light/8 h dark cycle at 22 °C for one week. The fungal isolate deposited in our laboratory collection, was stored at − 80 °C until use [66].

### Isolation of *P. xanthii* haustorial cells

The haustorial cells were isolated from zucchini cotyledons 10 days after inoculation with *P. xanthii* as previously described [5] with minor modifications: after homogenization, a 40 μm nylon mesh was used. The resulting homogenate was treated with 0.025 mg/ml wheat germ agglutinin (WGA)-Alexa Fluor 488 conjugate (Thermo Fisher Scientific, Waltham, MA, USA) in PBS to label the haustorial cell walls. Fluorescence-activated cell sorting was used to separate the haustoria from other fungal structures, plant organelles and debris. For this, a Beckman Coulter MoFlo flow cytometer (Beckman Coulter, Brea, CA, USA) was employed. Cells were sorted at a rate of approximately 5000 events per sec and 25 psi of pressure. The homogenate was agitated during sorting to avoid cell aggregation. Forward scatter and green fluorescence parameters of the particles in the homogenate were used upon excitation with a laser emitting 125 mW at 48 nm to separate the haustorial cells. Green fluorescence and a dot plot of green fluorescence vs forward scatter were used to adjust electronic gating to select those particles with high green fluorescence and forward scatter properties that corresponded to a size between 15 μm – 20 μm, which is equivalent to the size of the *P. xanthii* haustorium [5]. Preliminary assays to optimize threshold settings for the forward scatter were necessary. The initial homogenate and the final elution from flow cytometry were examined by confocal laser scanning microscopy (CLSM) in a Leica SP5 II confocal microscope (Leica Microsystems, Wetzlar, Germany) to determine the correct separation of collected cells. All steps, including the flow cytometry, were performed at 4 °C to avoid RNA degradation.

### RNA extraction, cDNA library synthesis and sequencing

Total RNA extraction from isolated haustoria was performed immediately after cell sorting using a PicoPure RNA isolation kit (Arcturus Bioscience, Mountain View, CA USA) according to the manufacturer’s instructions. Total RNA was eluted in DEPC-treated water and stored at − 80 °C until use. The quantity and quality of the total RNA was measured by an Agilent 2100 bioanalyser using an RNA Pico 6000 chip (Agilent Technologies, Santa Clara, CA, USA). cDNA and Illumina library synthesis was carried out using the Ovation Universal RNA-Seq System (NuGEN Technologies, San Carlos, CA, USA) according to the manufacturer’s instructions. First strand synthesis of total RNA was carried out employing an oligo (dT) primer mix and a random primer mix. For library construction, cDNA fragmentation was performed using NEBNext dsDNA Fragmentase (New England Biolabs, Ipswich, MA, USA) for 30 min at 37 °C. The Illumina adapters were then added according to the manufacturer’s instructions. The ribosomal 18S and 28S sequences of *P. xanthii* (MK225523 and MK225524, respectively) were used to design custom InDA-C primers (Additional file 1: Table S10), which were necessary to deplete ribosomal sequences from the total RNA sample using the Ovation Universal RNA-Seq System (NuGEN Technologies Inc.). The libraries were then sequenced on an Illumina NextSeq 550 instrument (Illumina, San Diego, CA, USA) using the read layout 2 × 150 nt.

### De novo assembly and annotation of transcriptomes

The Illumina raw reads from haustorial RNA and 975,070 Roche 454 raw single-end reads from hyphal and conidial RNA previously published by our laboratory [25] were used to perform the *P. xanthii* transcriptomic profile. This profile comprises the haustorial transcriptome and the revised version of the epiphytic transcriptome, produced in order to compare it with the haustorial transcriptome, generated using the TransFlow workflow as described in our previous study [27]. TransFlow is a modular framework designed for 454 Roche and Illumina reads that i) pre-processes raw reads, ii) builds several tentative transcriptomes and iii) chooses the best transcriptome. The workflow was executed using the Module 1 for Illumina data, the Module 2 for the 454 Roche data, the Module 4 for assessing the reference transcriptomes and the Module 5 for assessing the putative transcriptomes and select the best candidates. Transcriptomes of *Neurospora crassa* (SRR100067) and *Candida albicans* (SRR2005826) were downloaded from ENSEMBL release 31 and used as reference transcriptomes for evaluation in Module 4. Finally, the best epiphytic (Epi) and haustorial (Hau) transcriptomes from all assemblies produced were selected according to their comparison with the reference transcriptomes. The selected transcriptomes were full annotated with orthologues in Kingdom Fungi from UniProtKB using Full-LengtherNext (http://www.scbi.uma.es/fulllengthernext).

### Characterization of the *P. xanthii* haustorial transcriptome

To reveal the specific functions of the haustorium, a comparison between the epiphytic and haustorial transcriptomes was carried out using the GO terms that were associated with all unigenes from both transcriptomes. In this way, GO terms retrieved from the Full-LenghterNext annotation results were compared by Venn diagram calculation performed using Venny web-based software (http://bioinfogp.cnb.csic.es/tools/venny/). Only those GO terms that were present in the haustorial transcriptome were selected. To avoid redundant terms, to decrease the complexity and to obtain a visual representation of specific haustorial GO terms, REVIGO web-based software [67] with a medium (0.7) allowed similarity parameter was used. REVIGO reduction analysis tool allows to condense the GO description by removing redundant terms and to cluster the closer GO terms in a two dimensional space. In addition, the top 50 most highly expressed genes in the haustorial cDNA library of *P. xanthii* were calculated by aligning the trimmed reads with respect to all contigs obtained in the assembly process. This process was carried out using Bowtie2 [68]. GO terms related to biological processes associated with these top 50 most highly expressed genes were obtained from Full-LengtherNext annotation results and visually represented by REVIGO with a medium (0.7) allowed similarity parameter.

On the other hand, due the high number of ncRNAs that were present in the haustorial transcriptome according to Full-LenghterNext annotation, and in order to avoid the possible artefacts, the trimmed reads were also aligned against all ncRNAs and only those with 10 or more mapping reads were selected. Then, an analysis of the top 50 expressed ncRNAs was performed as described above. Those ncRNAs were annotated with the non-coding RNA sequence database RNAcentral (https://rnacentral.org/), which includes 31 databases of ncRNA. Finally, the expression pattern of some of these ncRNAs during the first steps of infection was investigated by qRT-PCR as described below.

### Definition of predicted secretomes and selection of specific haustorial secreted proteins

To predict the panel of secreted proteins with signal peptide produced by the epiphytic structures and the haustoria of *P. xanthii*, two web-based software programs were used. Both epiphytic and haustorial secretomes were defined using the combination of Secretool [69] and PECAS (Prokaryotic and Eukaryotic Classical Analysis of Secretomes) software [70]. Both Secretool and PECAS are prediction pipelines that comprise a group of web tools (SignalP, TargetP among others), allowing to predict a complete secretome in a single step from amino acid sequence files. To reproduce the parameters used to predict the epiphytic secretome previously described [25], the parameters used were the following: 0.45 SignalP cut-off probability, 0 Target P cut-off probability, 1 maximum transmembrane domain and a 17 WoLF PSORT cut-off score for Kingdom Fungi. Later, to perform a more accurate prediction of candidate secreted proteins and avoid false positives, the DeepLoc web-based software [71] was used. DeepLoc is a novel bioinformatics tool that allows predicting the subcellular localization of proteins, even those without annotated homologues, using deep learning. In addition, to discern the secreted proteins only expressed in haustoria, the UniProtKB protein identifiers of epiphytic and haustorial candidate secreted proteins were obtained from Full-LengtherNext annotation results and compared by Venn diagram. In this way, a list containing *Podosphaera* haustorium-specific secreted proteins was obtained.

### Protein structure modelling and protein function prediction

To gain insight into the putative functions of the *Podosphaera* haustorial effector candidates (PHECs), the website I-TASSER (http://zhanglab.ccmb.med.umich.edu/I-TASSER/) [72] was used to perform automated protein structure homology modelling by fold recognition searches using crystal structure of proteins with known functions available in the Protein Data Bank (PDB). To measure the quality of a predicted structure, its estimated TM (template modelling) score and C (confidence) score values were used. According to I-TASSER, TM score values higher than 0.5 indicate the correct topology, and values lower than 0.17 indicate a random similarity. A C score value in the range between − 5 and 2 indicates a more confident model.

The website Phyre2 (http://www.sbg.bio.ic.ac.uk/phyre2/html/page.cgi?id=index) [73] was used to carry out the search for analogous structures. Additional software tools used to obtain more information about the putative functions or domains of PHECs were CATH/Gene3D (http://www.cathdb.info/search/by_sequence) [74] and MotifScan (https://myhits.isb-sib.ch/cgi-bin/motif_scan#prf:SER_RICH) [75].

### PCR and qRT-PCR

To validate their exclusive or preferential expression in haustoria of genes initially classified as coding for haustorium-specific secreted proteins, a PCR analysis using cDNA obtained from epiphytic and haustorial structures was carried out as previously described [24]. For this purpose, haustorial cells were isolated from infected zucchini cotyledons at 10 days after inoculation with *P. xanthii* and total RNA was extracted as described above*.* Similarly, spores and hyphae were removed carefully from infected zucchini cotyledons at 10 days after inoculation with the pathogen and total RNA was isolated using TRI Reagent (Sigma-Aldrich, Saint Louis, MO, USA) according to the manufacturer’s indications. The synthesis of cDNA was performed using total RNA, random primers and Superscript III Reverse Transcriptase (Thermo Fisher Scientific, Waltham, MA, USA) according to the manufacturer’s instructions. PCR was carried out using the primer pairs listed in (Additional file 1: Table S11). PCR conditions were identical to those described previously [24].

For the quantification of the expression of ncRNAs and haustorial-specific secreted protein coding genes during the early stages of infection, qRT-PCR analysis was performed. For this analysis, zucchini cotyledons inoculated with *P. xanthii* were collected at 0, 24, 48 and 72 h post-inoculation, frozen in liquid nitrogen and ground with a mortar and pestle. Total RNA extractions were carried out using TRI Reagent (Sigma-Aldrich) and cDNA synthesis was perfomed as described above. SsoFast EvaGreen Supermix (Bio-Rad, Hercules, CA, USA) was used to perform the qRT-PCR reactions according to the manufacturer’s instructions, in a CFX384 Touch Real-Time PCR detection system (Bio-Rad) and with the primer pairs listed in (Additional file 1: Table S11). The qRT-PCR conditions were as follows: enzyme activation step at 95 °C for 30 s, followed by 40 cycles of 5 s at 95 °C and 5 s at 65 °C. After amplification, the data were analysed using CFX Manager software (Bio-Rad). Additionally, the amplicon size was confirmed by visualization on 2% agarose gels. All primers used in PCR and qRT-PCR analyses were designed using Primer3 [76]. Furthermore, a clustered heat map of expression of the genes using correlation distance and average linkage was performed using ClustVis [77].

## Additional files

Additional file 1: **Table S1.** Summary of best epiphytic and haustorial transcriptomes generated by TransFlow. These assemblies comprise primary assemblies of 454 Roche and Illumina reads generated with several assemblers and different combinations of them. **Table S10.** Customized InDA-C primers used to deplete ribosomal sequences from total RNA extractions during the process of cDNA library synthesis. **Table S11.** Primers used in this study for PCR and qRT-PCR analyses. (PDF 296 kb)

Additional file 2: **Table S2.** Full annotation of *P. xanthii* haustorial and epiphytic transcriptomes. **Table S3.** Comparison of haustorial and epiphytic GO terms performed by Venn diagram. **Table S4.** Non-redundant GO terms related to biological processes specific to *P. xanthii* haustoria obtained by REVIGO. **Table S5.** Top 50 expressed unigenes in *P. xanthii* haustoria. **Table S6.** Non-redundant GO terms related to biological processes of the top 50 expressed haustorial unigenes obtained by REVIGO. **Table S7.** Top 50 expressed ncRNAs in *P. xanthii* haustorial transcriptome. **Table S8.** Subcellular localization analysis of haustorial and epiphytic candidate secreted proteins of *P. xanthii* performed by DeepLoc. **Table S9.** Annotation of *P. xanthii* haustorial and epiphytic secretomes. (XLSX 5574 kb)

## Data Availability

The datasets generated and analyzed during the current study are available in the SRA NCBI repository at BioProject PRJNA393391 (SRX5651176, SRX5645704).
