# Supplementary material for: The haustorial transcriptome of the cucurbit pathogen Podosphaera xanthii reveals new insights into the biotrophy and pathogenesis of powdery mildew fungi
Source: BMC Genomics. 2019 Jul 4;20:543. doi: 10.1186/s12864-019-5938-0 (PMC6611051; doi:10.1186/s12864-019-5938-0)
Supplement: Supplementary file 1 — Table S1. Summary of best epiphytic and haustorial transcriptomes generated by TransFlow. These assemblies comprise primary assemblies of 454 Roche and Illumina reads generated with several assemblers and different combinations of them. Table S10. Customized InDA-C primers used to deplete ribosomal sequences from total RNA extractions during the process of cDNA library synthesis. Table S11. Primers used in this study for PCR and qRT-PCR analyses. (PDF 296 kb) [file 12864_2019_5938_MOESM1_ESM.pdf]

Table S1. Summary of best epiphytic and haustorial transcriptomes generated by TransFlow. These assemblies comprise primary assemblies of 454 Roche and Illumina reads generated with several assemblers and different combinations of them.

**Structure**	**Assembly ID**	**Assembly description**[a]	**MD**[b]
Haustorium	scOases_cat_cd_rcMin2	Concatenation of two OASES assemblies with different *k*mers and sequence redundancy removal and reconciliation	0.3848
	scOases_cat	Concatenation of two OASES assemblies with different *k*mers	0.3850
	scOases_cat_cd	Concatenation of two OASES assemblies with different *k*mers and sequence redundancy removal and reconciliation	0.3861
	scSoap_cat_cd	Concatenation of two SOAP assemblies with different *k*mers and sequence redundancy removal and reconciliation	0.3887
	scOasesK35	OASES assembly with *k*-mer 35	0.3915
Epiphytic structures	ctMIRA_ctEulK29_rcCAP3	Primary assemblies (MIRA4 and EULER-SR) reconciliation using CAP3	0.0558
	ctMIRA	MIRA4-primary assembly	0.0688
	ctEulK29	EULER-SR primary assembly with *k*-mer 29	0.1435

[a]Summarized from Seoane et *al.* 2018.

[b]A Mean distance (MD) close to 0 means a high quality assembly and greater values show low quality assemblies.

Table S10 Customized InDA-C primers used to deplete ribosomal sequences from total RNA extractions during the process of cDNA library synthesis.

rRNA[a]	Primer name	Sequence	Position[b]
5.8S	1_5,8S_0	5'-GATGAAGAACGCAGCGAAAT-3'	0
	2_5,8S_70	5'-TCGAATCTTTGAACGCACATT-3'	70
18S	3_18S_0	5'-GGGGCCAGTCCGAAAG-3'	0
	4_18S_70	5'-TACAAAGGGCAGGGACGTAA-3'	70
	5_18S_140	5'-CAATGCTCTATCCCCAGCAC-3'	140
	6_18S_210	5'-AGAAACTCGTTGGCTCTGTCA-3'	210
	7_18S_280	5'-ATTGCCTCAAACTTCCATCG-3'	280
	8_18S_350	5'-CCTGGCTATTTAGCAGGTTAAGG-3'	350
	9_18S_420	5'-AACTAAGAACGGCCATGCAC-3'	420
	10_18S_490	5'-TCTGGACCTGGTGAGTTTCC-3'	490
	11_18S_560	5'-GCCCTTCCGTCAATTTCTTT-3'	560
	12_18S_630	5'-GTAAGGTGCCGAGCGAGTC-3'	630
	13_18S_700	5'-TGATCGTCTTCGATCCCCTA-3'	700
	14_18S_770	5'-CATCCTTGGCAAATGCTTTC-3'	770
	15_18S_840	5'-CATTACGGCGGTCCTAGAAA-3'	840
	16_18S_910	5'-GAGCATAGGCCTGCTTTGAA-3'	910
	17_18S_980	5'-AGTCCTGGTTCCCCGACAC-3'	980
	18_18S_1050	5'-AGGCCCAAGGTTCAACTACG-3'	1050
	19_18S_1120	5'-TTTAATATACGCTATTGGAGCTGGA-3'	1120
	20_18S_1190	5'-CCCTCCAATTGTTCCTCGTT'-3'	1190
	21_18S_1260	5'-TTGTCACTACCTCCCCGTGT-3'	1260
	22_18S_1330	5'-GTTTCTCAGGCTCCCTCTCC-3'	1330
	23_18S_1400	5'-GGGCAGAAATTTGAATGAACC-3'	1400
	24_18S_1470	5'-GCCATGCGATTCGTTAAGTT-3'	1470
	25_18S_1540	5'-GAAGTCGGGGCTTTTAGCAT-3'	1540
	26_18S_1610	5'-AATGAGCCATTCGCAGTTTC-3'	1610
28S	27_28S_0	5'-ACCTCAGTAACGGCGAGTGA-3'	0
	28_28S_70	5'-GGCCTGGCCTAAGTTCCTT-3'	70
	29_28S_140	5'-GGACGTCGTAGAGGGTGAGA-3'	140
	30_28S_210	5'-GTCGAGTTGTTTGGGAATGC-3'	210
	31_28S_280	5'-AGCGCACAAGTAGAGTGATCG-3'	280
	32_28S_350	5'-TGATCCGCTAGGGTTCTCTCT-3'	350
	33_28S_420	5'-AGAATGGCTGGTGGAATGTG-3'	420
	34_28S_490	5'-CGCTTCGGCTAGGATGC-3'	490
	35_28S_560	5'-TGAAACACGGACCAAGGAGT-3'	560
	36_28S_630	5'-TAAACCCATACGCGGAATGA-3'	630
	37_28S_700	5'-GGACCCGAAAGATGGTGAAC-3'	700
	38_28S_770	5'-CCAGAGGAAACTCTGGTGGA-3'	770
	39_28S_840	5'-CCGAAGTTTCCCTCAGGATAG-3'	840
	40_28S_910	5'-ATTAGAGGCCTTGGGGTTGA-3'	910
	41_28S_980	5'-CCTTGTTACTTAATTGAACGTGGAC-3'	980
	42_28S_1050	5'-GGCCATTTTTGGTAAGCAGA-3'	1050
	43_28S_1120	5'-ACGCTCATCAGACACCACAA-3'	1120
	44_28S_1190	5'-CCGAATGAACTAGCCCTGAA-3'	1190
	45_28S_1260	5'-TACTTCACCGCCAGGGTAGA-3'	1260
	46_28S_1330	5'-GTCGAACGGCCTCTAGTGC-3'	1330
	47_28S_1400	5'-GAAGTGGGGAAAGGTTCCAT-3'	1400
	48_28S_1470	5'-CGAAAGGGAAGCCGGTTAAT-3'	1470
	49_28S_1540	5'-ATTCTCCACGGCAACGTAAC-3'	1540
	50_28S_1610	5'-CACCCTGAAATCGGTTTGTC-3'	1610
	51_28S_1680	5'-GCACCTTTGTCAGGTCTGGT-3'	1689
	52_28S_1750	5'-ACTCATAACCGCAGCAGGTC-3'	1750
	53_28S_1820	5'-AACTTCGGGAAAAGGATTGG-3'	1820
	54_28S_1890	5'-AGGTCGCCACTAGCCTCAC-3'	1890
	55_28S_1960	5'-CCGGCGTACAATTAACAACC-3'	1960
	56_28S_2030	5'-GGCCAGAAAGTGGTGTTGAC-3'	2030
	57_28S_2100	5'-TTCTGCCCAGTGCTCTGAAT-3'	2100
	58_28S_2170	5'-ACGCGCATGAATGGATTAAC-3'	2170
	59_28S_2240	5'-CTAGCGAAACCACAGCCAAG-3'	2240
	60_28S_2310	5'-AAGACCCTGTTGAGCTTGACTCT-3'	2310
	61_28S_2380	5'-TTATTCAATGAAGCGGAGCTG-3'	2380
	62_28S_2450	5'-GATCCGGGTTGAAGACATTG-3'	2450
	63_28S_2520	5'-GGGGACTCATGGAGAACAGA-3'	2520
	64_28S_2590	5'-GTCCCCTTGATTTTGATTTTCA-3'	2590
	65_28S_2660	5'-GGTGCCAGAAAAGTTACCACA-3'	2660
	66_28S_2730	5'-CGACGTTGCTTTTTGATCCT-3'	2730
	67_28S_2800	5'-CGGTAAGCGTTGGATTGTTC-3'	2800
	68_28S_2870	5'-CCGCAATGGTAATTCAGCTT-3'	2870
	69_28S_2940	5'-TAATTGGTTTTTGCGGCTGT-3'	2940

[a] Ribosomal RNA subunit depleted.
[b] Primer position into rRNA molecule.

Table S11 Primers used in this study for PCR and qRT-PCR analyses.

Unigene/ncRNA	Primer name	Sequence	Amplicon size (bp)
689	689-F	5’-AGGACTGATGGAAGCGAAGA-3’	158
	689-R	5’-GAGCCGCGTAGAACAGTTTC-3’	
15314	15314-F	5’-TGACGATCAATCACGCTCTC-3’	158
	15314-R	5’-AGGTTCCGCAGCTTTCACTA-3’	
15509	15509-F	5’-CTCACCGGAGCAAGCTAGAC-3’	219
	15509-R	5’-AGCGCGTCAAGAAGATGTTT-3’	
15569	15569-F	5’-CTCGGCTTCTTTGGATCAAC-3’	230
	15569-R	5’-AGTCTCTTGGTTGCGGAAGA-3’	
15584	15584-F	5’-ATGGCTCAGTGCAGACAGTG-3’	211
	15584-R	5’-CACCGGGTTTAACTTCCAGA-3’	
15629	15629-F	5’-CCACTCGAGACCGTGGTAAT-3’	201
	15629-R	5’-CGATTCAGGGGAGTACAGGA-3’	
15673	15673-F	5’-GCTCCAGCGAAACTTACAGG-3’	193
	15673-R	5’-AAACCTCAACGCTCTCTCCA-3’	
15694	15694-F	5’-GTCCCGGTGAGACTGTTGAT-3’	172
	15694-R	5’-GGATCGGTGAAAGCAATGTT-3’	
27213	27213 -F	5’-AGTGTGCACCAATTCCACTG-3’	154
	27213-R	5’-TCCCATAACAATTGCACCAA-3’	
217529	217529-F	5’-ATATGCTGTTGCTGCTGTCG-3’	261
	217529-R	5’-TACAGCATCCAGTCCACCAG-3’	
15544	15544-F	5’-CGAATCCACCGAGATCCTTA-3’	169
	15544-R	5’-TTTCAACCCTTTCTCGGTTG-3’	
223421	223421-F	5’-AATGTGGCTCTACGGGATTG-3’	242
	223421-F	5’-GCAGAGATACCTCGGTGGAA-3’	
nc11307	11307-F	5’-GAAGTGATTGGGCCAGTTGT-3’	243
	11307-R	5′-TCGTAAGCCCCGTTCTGTTA-3’	
nc17753	17753-F	5’-TTAAGGTGGTTGGAGGTTGG	249
	17753-R	5’-CAGGCTGAGGACTACCCATC	
nc15465	15465-F	5’-TCGAACGCACACAAAACATT	186
	15465-R	5’-ACCAGGTCGAGTTGAGAACG	
nc7919	7919-F	5’-TATCCTTCCACCGACAGGAG	185
	7919-R	5’-GAGATTGGTTCAGCCCATTG-3’	
nc13870	13870-F	5’-GCATCAGAAAAGCAGGACAA-3’	164
	13870-R	5’-ACTTGCTTAGGCTGCGAAAA-3’	
nc1955	1955-F	5’-CCCAGTAGTCATTCAGGAGCA-3’	197
	1955-R	5’-CCACACCACACCTATTCCAA-3’	
